# JMJD1A Represses the Development of Cardiomyocyte Hypertrophy by Regulating the Expression of *Catalase*

**DOI:** 10.1155/2020/5081323

**Published:** 2020-05-12

**Authors:** Rongjia Zang, Qingyun Tan, Fanrong Zeng, Dongwei Wang, Shuang Yu, Qingdong Wang

**Affiliations:** ^1^Department of Anesthesiology, First Affiliated Hospital of Jiamusi University, Heilongjiang Province, China; ^2^Department of Cardiology, First Affiliated Hospital of Jiamusi University, Heilongjiang Province, China

## Abstract

The histone demethylase JMJD family is involved in various physiological and pathological functions. However, the roles of JMJD1A in the cardiovascular system remain unknown. Here, we studied the function of JMJD1A in cardiac hypertrophy. The mRNA and protein levels of *JMJD1A* were significantly downregulated in the hearts of human patients with hypertrophic cardiomyopathy and the hearts of C57BL/6 mice underwent cardiac hypertrophy induced by transverse aortic constriction (TAC) surgery or isoproterenol (ISO) infusion. In neonatal rat cardiomyocytes (NRCMs), siRNA-mediated *JMJD1A* knockdown facilitated ISO or angiotensin II-induced increase in cardiomyocyte size, protein synthesis, and expression of hypertrophic fetal genes, including *atrial natriuretic peptide* (*Anp*), *brain natriuretic peptide* (*Bnp*), and *Myh7*. By contrast, overexpression of *JMJD1A* with adenovirus repressed the development of ISO-induced cardiomyocyte hypertrophy. We observed that *JMJD1A* reduced the production of total cellular and mitochondrial levels of reactive oxygen species (ROS), which was critically involved in the effects of JMJD1A because either N-acetylcysteine or MitoTEMPO treatment blocked the effects of *JMJD1A* deficiency on cardiomyocyte hypertrophy. Mechanism study demonstrated that JMJD1A promoted the expression and activity of *Catalase* under basal condition or oxidative stress. siRNA-mediated loss of *Catalase* blocked the protection of JMJD1A overexpression against ISO-induced cardiomyocyte hypertrophy. These findings demonstrated that JMJD1A loss promoted cardiomyocyte hypertrophy in a Catalase and ROS-dependent manner.

## 1. Introduction

Epigenetic regulation and posttranslational regulation of histone and nonhistone proteins are critically involved in the development of cardiac hypertrophy [1–3]. The histone deacetylases essentially participate in the development of cardiac hypertrophy by regulating the metabolism, mitochondrial homeostasis, and gene transcription [4–8]. In comparison to histone acetylation, the roles of histone methylation enzymes in cardiac hypertrophy are largely unknown. Lysine methylation is one of the most prominent histone posttranslational modifications that regulate chromatin structure and gene expression. Changes in histone lysine methylation status have been observed during cancer formation and development, which is a consequence of the dysregulation of histone lysine methyltransferases or demethylases [9, 10]. Recent studies have implicated the roles of histone methylation/demethylation in cardiac hypertrophy and fibrosis [10, 11].

The JMJD (JmjC domain-containing) proteins family is composed of 30 members in humans based on the presence of the roughly 150 amino acid–long JmjC domain [12]. One of the largest JMJD subfamilies that has recently attracted much attention is the JMJD2 proteins (JMJD2A-JMJD2D), which are capable of recognizing di- and trimethylated H3K9 and H3K36 as well as trimethylated H1.4K26 as substrates [9]. The most studied member of the JMJD2 family may be JMJD2A. A major study focusing on JMJD2A has been in transcription regulation, where it may either stimulate or repress gene transcription. JMJD2A functions in human Wiskott-Aldrich syndrome [13], Kaposi's sarcoma-associated herpesvirus replication [14], cardiac hypertrophy [15], and DNA repair [16]. For instance, JMJD2A promotes cardiac hypertrophy in response to hypertrophic stimuli in mice through binding to the FHL1 promoter, upregulating FHL1 expression, and downregulating H3K9 trimethylation [15].

JMJD1A is another member of this family. The roles of JMJD1A in tumor biology are widely studied. For instance, JMJD1A promotes alternative splicing of AR variant 7 (AR-V7) in prostate cancer cells [17]. JMJD1A regulates the transcriptional program of the androgen receptor in prostate cancer cells [18]. JMJD1A also promotes urinary bladder cancer progression by enhancing glycolysis through the coactivation of hypoxia-inducible factor 1*α* [19]. In addition, JMJD1A promotes cell growth and progression *via* transactivation of c-Myc expression and predicts a poor prognosis in cervical cancer. JMJD1A was also reported to participate in thermogenesis [20]. Regulation of c-Myc expression by the histone demethylase JMJD1A is essential for prostate cancer cell growth and survival [21]. A previous study revealed the participation of JMJD1A in cardiac hypertrophy, but the underlying mechanisms are not fully understood [22]. In this study, we aimed at investigating the potential function and mechanism of JMJD1A in cardiac hypertrophy.

## 2. Materials and Methods

### 2.1. Patients

Human heart samples were obtained from the First Affiliated Hospital of Jiamusi University transplant program. Control samples were obtained intraoperatively from nonfailing hearts undergoing ventricular corrective surgery. Failing heart specimens were obtained from diseased hearts that were removed during orthotopic heart transplantation. Informed consent was obtained from all patients participating in this study. All procedures involving human tissue use were approved by the Ethics Review Board of the First Affiliated Hospital of Jiamusi University.

### 2.2. Experimental Animal Models of Cardiac Hypertrophy

8-12 weeks old C57BL/6 mice were subjected to TAC surgery for 28 days to induce cardiac hypertrophy. The control mice were undergoing sham surgery. ISO (Sigma-Aldrich) was dissolved in 150 mM NaCl and 1 mM acetic acid, and they were delivered (8.7 mg/kg/d for 28 days) by implanting of Osmotic Minipumps (model 2004; ALZET) into the abdomens of adult mice. Control mice underwent the same procedure, except that the respective pumps were filled only with vehicle (150 mM NaCl and 1 mM acetic acid). The development of hypertrophy was judged noninvasively by the use of echocardiography. The animal study was approved by the Ethics Review Board of Animal Study at the First Affiliated Hospital of Jiamusi University.

### 2.3. Isolation and Culture of Neonatal Rat Cardiomyocytes

Neonatal rat cardiomyocytes (NRCMs) were cultured and infected with adenoviral vectors as described earlier [23]. The NRCMs were cultured in DMEM supplemented with 10% fetal bovine serum (Thermo Fisher), 0.1 mM 5-bromodeoxyuridine (to inhibit fibroblast proliferation), and 1% penicillin/streptomycin (Invitrogen). The animal study was approved by the Ethics Review Board of Animal Study at the First Affiliated Hospital of Jiamusi University.

### 2.4. Packaging of Adenovirus

Replication-defective adenoviral vectors expressing rat *Jmjd1a* (Ad-*Jmjd1a*) or control green fluorescent protein (Ad-Ctrl) were generated using the AdEasy Vector kit (Quantum Biotechnologies) according to previously described methods.

### 2.5. siRNA Transfection

For knocking down the expression of *Jmjd1a* or *Catalase*, siRNA was synthesized and transfected into NRCMs with the RNAiMax transfection kit (Invitrogen). The siRNA sequence was as follows. si*Jmjd1a-1*# 5′- GCACAGTCCTCCATACGTT-3′, si*Jmjd1a-2*# 5′- GGAUGUAAACAGUCUUCGA-3′, si*Catalase*: 5′- CCAGAUACUCCAAGGCAAATT-3′.

### 2.6. Western Blot

Total protein was extracted from NRCMs or heart tissues with RIPA and protease inhibitors. Then, the protein was subjected to western blot as described previously [24]. The following antibodies were used: anti-JMJD1A antibody (Abcam, #ab106456), anti-*β*-Actin antibody (Cell Signaling Technology, #4967), anti-Catalase antibody (Santa Cruz, #sc-271803), anti-H3K9me1 antibody (Abcam, #ab9045), anti-H3K9me2 antibody (Abcam, #ab1220), and anti-Histone H3 antibody (Abcam, #ab8898).

### 2.7. Quantitative Real-Time PCR (qRT-PCR)

Total cellular RNA was isolated from cardiomyocytes or heart tissues using the TRIzol Reagent (Invitrogen). One microgram of DNase-I–treated RNA was reverse transcribed using the SuperScript III kit (Invitrogen). The resultant cDNA was subjected to quantitative real-time PCR with SYBR GREEN II (TAKARA). The primers used for qRT-PCR are listed in the Supplementary data (available here).

### 2.8. Induction of Cardiomyocyte Hypertrophy

NRCMs were cultured for 48 hours, then infected with indicated adenovirus or transfected with siRNA and cultured in serum-free medium for 24 hours. Next, the cells were treated with ISO (1 *μ*M) or angiotensin II (Sigma, #A9525, 1 *μ*M) for 48 hours in serum-free medium to induce cardiomyocyte hypertrophy.

### 2.9. Cardiomyocyte Size Analysis

Then, cardiomyocytes were stained with *α*-actinin antibody (Siamg, #A7811). The cell size of cardiomyocytes was measured by using NIH ImageJ software (http://rsbweb.nih.gov/ij/). More than 100 cells were quantified per experiment, and the average values of three independent experiments were used for quantification.

### 2.10. Protein Synthesis Analysis

The hypertrophic response of cells was measured by [^3^H]-leucine incorporation into total protein as described previously [25].

### 2.11. Measurement of Total and Mitochondrial ROS Level

Total ROS and mitochondrial ROS levels were measured with the DHE staining kit or mitoSOX staining kit prospectively as described previously [8]. The relative ROS levels were monitored and evaluated with image J.

### 2.12. Catalase Activity Assay

The activity of Catalase in cardiomyocytes was monitored with Catalase Activity Assay Kit (Abcam, # ab83464).

### 2.13. Statistical Analysis

All values are expressed as the mean ± S.D. Statistical differences among groups were determined using either Student's *t* test (for two groups) or one-way ANOVA (for more than two groups) using Graph-Pad Prism 8 Software. *p* values < 0.05 were considered significant.

## 3. Results

### 3.1. JMJD1A Expression Is Reduced during Cardiac Hypertrophy

**Figure 1:**
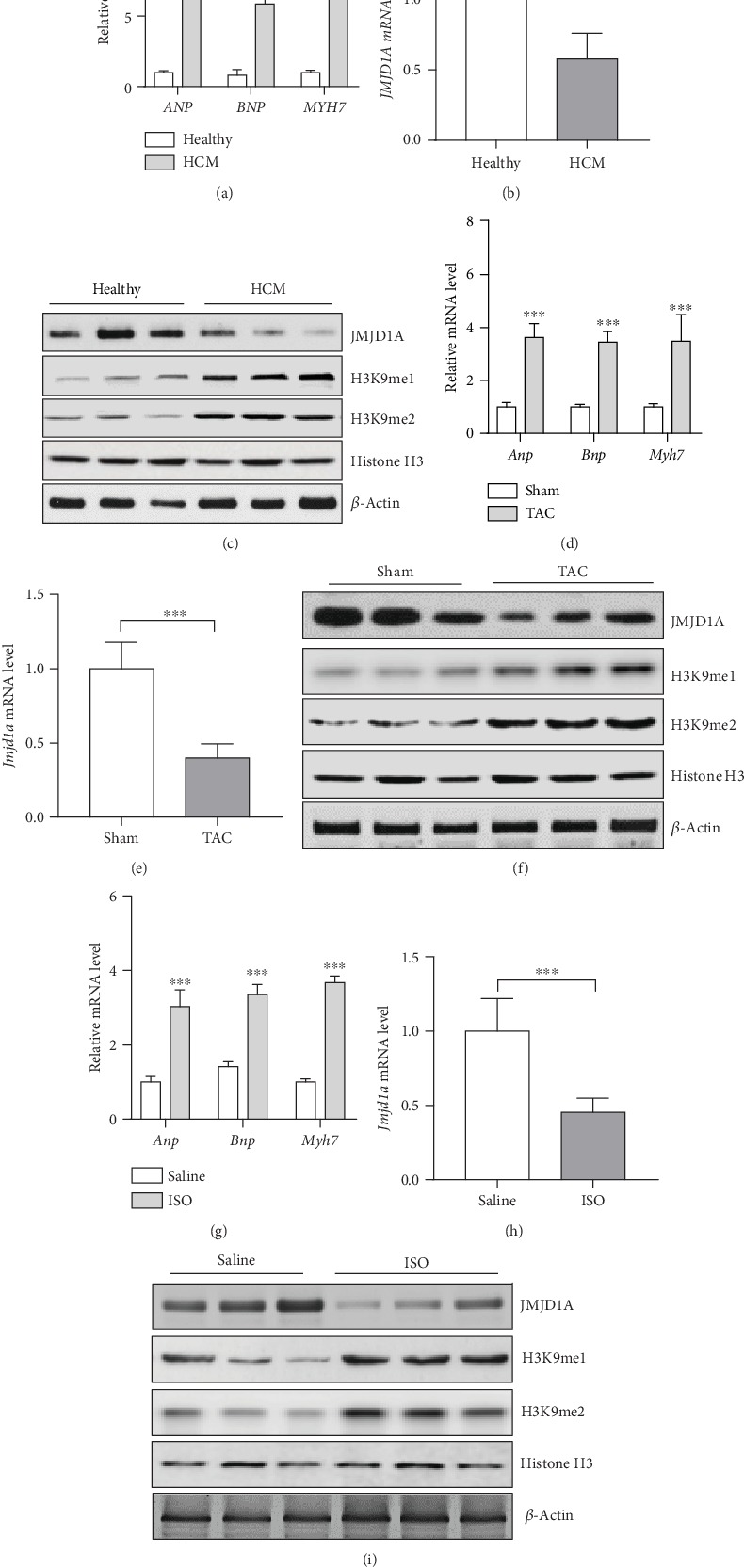
JMJD1A expression is downregulated during cardiac hypertrophy. (a) mRNA levels of hypertrophic fetal genes in the hearts from control or hypertrophic cardiomyopathy (HCM). ANP: atrial natriuretic peptide; BNP: brain natriuretic peptide; MYH7: beta-myosin heavy chain. *n* = 5 in each group, ^∗∗∗^*p* < 0.001*vs.* healthy group. (b) mRNA levels of *JMJD1A* in the hearts from control or hypertrophic cardiomyopathy (HCM). *n* = 5 in control group, *n* = 10 in HCM group, ^∗∗^*p* < 0.01. (c) Protein levels of JMJD1A and H3K9me1/2 in the hearts from control or hypertrophic cardiomyopathy (HCM). (d) mRNA levels of hypertrophic fetal genes in the hearts of mice underwent sham or TAC surgery. TAC: transverse aortic constriction. *n* = 5 in each group, ^∗∗∗^*p* < 0.001*vs.* sham group. (e) mRNA levels of *Jmjd1a* in the hearts of mice underwent sham or TAC surgery. *n* = 5 in each group, ^∗∗∗^*p* < 0.001*vs.* sham group. (f) Protein levels of JMJD1A and H3K9me1/2in the hearts from mice underwent sham or TAC surgery for four weeks. (g) mRNA levels of hypertrophic fetal genes in the hearts of mice underwent saline or ISO infusion for four weeks. *n* = 5 in each group, ^∗∗∗^*p* < 0.001*vs.* saline group. (h) mRNA levels of *Jmjd1a* in the hearts of mice underwent saline or ISO infusion for four weeks. *n* = 5 in each group, ^∗∗∗^*p* < 0.001*vs.* saline group. (i) Protein levels of JMJD1A and H3K9me1/2in the hearts from mice underwent saline or ISO infusion for four weeks.

The roles of JMJD1A in the cardiovascular system remain unknown; here, we aimed at investigating the potential roles of JMJD1A in cardiac hypertrophy. We first analyzed the expression of *JMJD1A* in hypertrophic hearts from patients and mice. We collected heart tissues from patients with hypertrophic cardiomyopathy (HCM, *n* = 10) and control healthy donors (*n* = 5). The mRNA levels of hypertrophic fetal genes were significantly upregulated in the hearts of HCM compared with the control donors (Figure 1(a)). We also analyzed the expression of *JMJD1A* in these samples and found that the mRNA level of *JMJD1A* was significantly downregulated in hypertrophic hearts of human (Figure 1(b)). In addition, we performed western blot to examine the protein level of JMJD1A. The results showed that the protein level of JMJD1A was significantly downregulated in the hearts of patients with HCM (Figure 1(c)). JMJD1A is a histone demethylase of H3K9me1/2. Indeed, we observed an increase in H3K9me1 and H3K9me2 in the HCM group (Figure 1(c)). Next, we analyzed the expression of *Jmjd1a* in mice with experimental cardiac hypertrophy. Cardiac hypertrophy was induced in C57BL/6 mice with TAC surgery. The expression of hypertrophic fetal genes was analyzed at four weeks post-TAC surgery. The results showed that the mRNA levels of hypertrophic fetal genes were remarkedly upregulated in TAC-induced hypertrophic hearts (Figure 1(d)). We next tested the expression of *Jmjd1a* mRNA and protein levels and found that either the mRNA or protein level of *Jmjd1a* was significantly downregulated in TAC-induced hypertrophic hearts (Figures 1(e) and 1(f)), which was accomplished with the increase in H3K9me1 and H3K9me2 (Figure 1(f)). In addition, we also induced cardiac hypertrophy in mice by infusing of ISO for four weeks. The ISO infusion significantly upregulated the expression of hypertrophic fetal genes in the hearts of mice (Figure 1(g)). Importantly, the results showed that ISO infusion downregulated the mRNA and protein levels of *Jmjd1a* in the hearts of mice infused with ISO for four weeks (Figures 1(h) and 1(i)), in couple with the upregulation of H3K9me1 and H3K9me2 (Figure 1(i)). Taken together, these findings demonstrated that the expression of JMJD1A was downregulated whereas H3K9me1 and H3K9me2 were increased in hypertrophic hearts of humans and mice.

### 3.2. JM9JD1A Represses the Development of Cardiomyocyte Hypertrophy

**Figure 2:**
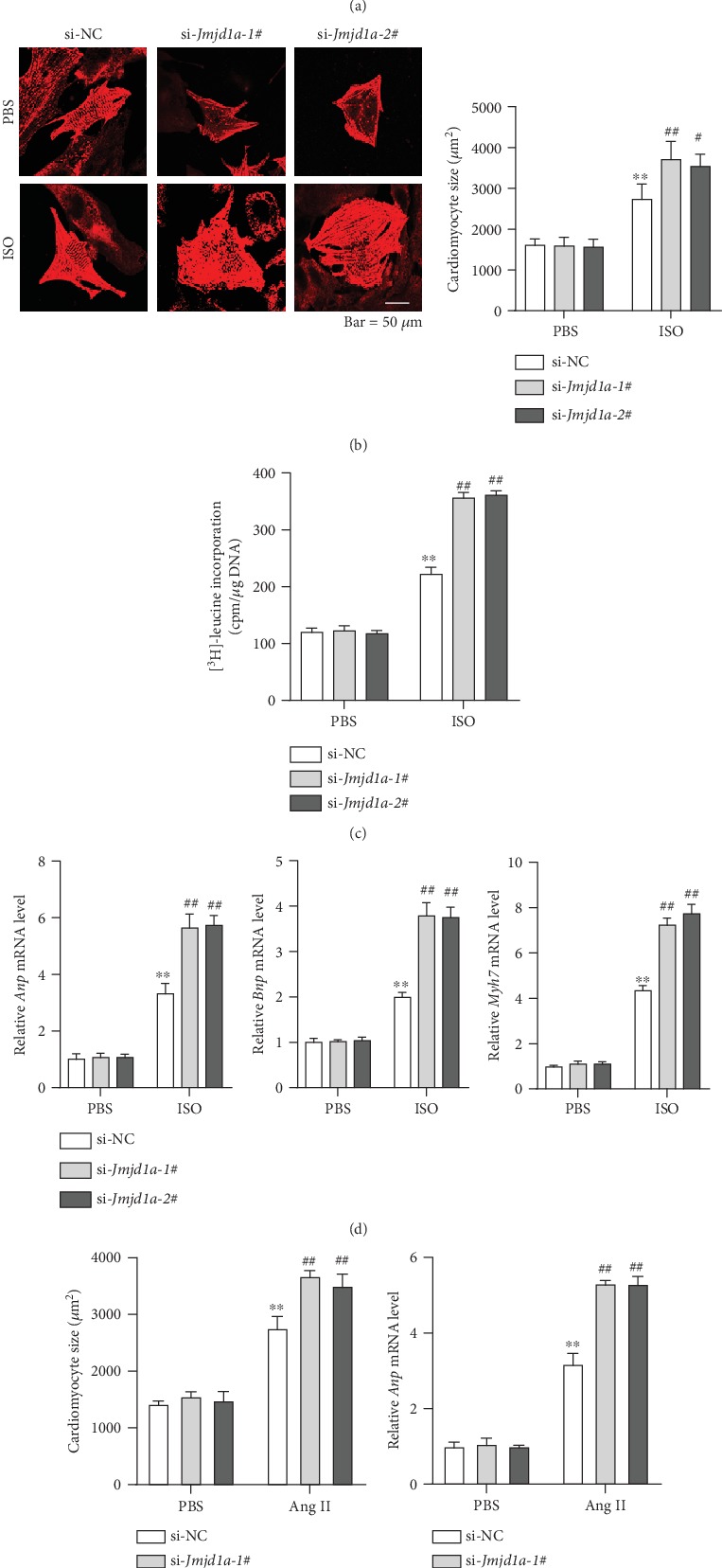
JMJD1A knockdown promotes cardiomyocyte hypertrophy of neonatal rat cardiomyocytes. (a) Representative western blot showing the expression of JMJD1A protein in neonatal rat cardiomyocytes transfected with si-*Jmjd1a* or si-NC. NRCMs were transfected with si-NC or si-*Jmjd1a* for 36 hours. (b) *Jmjd1a* knockdown promotes an ISO-induced increase in cardiomyocyte size. Representative images and quantitative results are shown. NRCMs were transfected with si-NC or si-*Jmjd1a* for 24 hours and then treated with ISO (1 *μ*M) for 48 hours. ^∗∗^*p* < 0.01*vs.* PBS+si-NC, #*p* < 0.05 and ^##^*p* < 0.01*vs.* ISO+si-NC. (c) *Jmjd1a* knockdown promotes ISO-induced protein synthesis in cardiomyocytes. NRCMs were transfected with si-NC or si-*Jmjd1a* for 24 hours and then treated with ISO (1 *μ*M) for 48 hours. ^∗∗^*p* < 0.01*vs.* PBS+si-NC, ^##^*p* < 0.01*vs.* ISO+si-NC. (d) *Jmjd1a* knockdown promotes ISO-induced expression of hypertrophic fetal genes. NRCMs were transfected with si-NC or si-*Jmjd1a* for 24 hours and then treated with ISO (1 *μ*M) for 48 hours. ^∗∗^*p* < 0.01*vs.* PBS+si-NC, ^##^*p* < 0.01*vs.* ISO+si-NC. (e) *Jmjd1a* knockdown promotes angiotensin II (Ang II)-induced increase in cardiomyocyte size. Representative images and quantitative results are shown. NRCMs were transfected with si-NC or si-*Jmjd1a* for 24 hours and then treated with Ang II (1 *μ*M) for 48 hours. ^∗∗^*p* < 0.01*vs.* PBS+si-NC, ^##^*p* < 0.01*vs.* Ang II+si-NC. (f) *Jmjd1a* knockdown promotes Ang II-induced expression of hypertrophic fetal genes. NRCMs were transfected with si-NC or si-*Jmjd1a* for 24 hours and then treated with Ang II (1 *μ*M) for 48 hours. ^∗∗^*p* < 0.01*vs.* PBS+si-NC, ^##^*p* < 0.01*vs.* Ang II+si-NC.

Next, we investigated whether the downregulation of JMJD1A during cardiac hypertrophy contributes to the development of cardiomyocyte hypertrophy. We isolated cardiomyocytes from the hearts of neonatal rats. Then, we knockdown the expression of *Jmjd1a* in neonatal rat cardiomyocytes (NRCMs) by transfecting the cells with siRNA (Figure 2(a)) and treated with the cells with ISO to induce cardiomyocyte hypertrophy. The results showed that ISO treatment significantly increased the size of NRCMs and *Jmjd1a* knockdown promoted the effects on ISO-induced increase in cardiomyocyte size (Figure 2(b)). Protein synthesis is a hallmark of cardiomyocyte hypertrophy. Indeed, ISO treatment induced the [^3^H]-leucine incorporation into cardiomyocytes and *Jmjd1a* knockdown promoted the effects of ISO (Figure 2(c)). We also tested the expression of hypertrophic fetal genes and found that *Jmjd1a* knockdown facilitated the expression of hypertrophic fetal genes (*Anp*, *Bnp*, and *Myh7*) induced by ISO (Figure 2(d)). We also induced cardiomyocyte hypertrophy with angiotensin II (Ang II). Similar results were obtained as evidenced by cardiomyocyte size and hypertrophic gene expression (Figures 2(e) and 2(f)).

**Figure 3:**
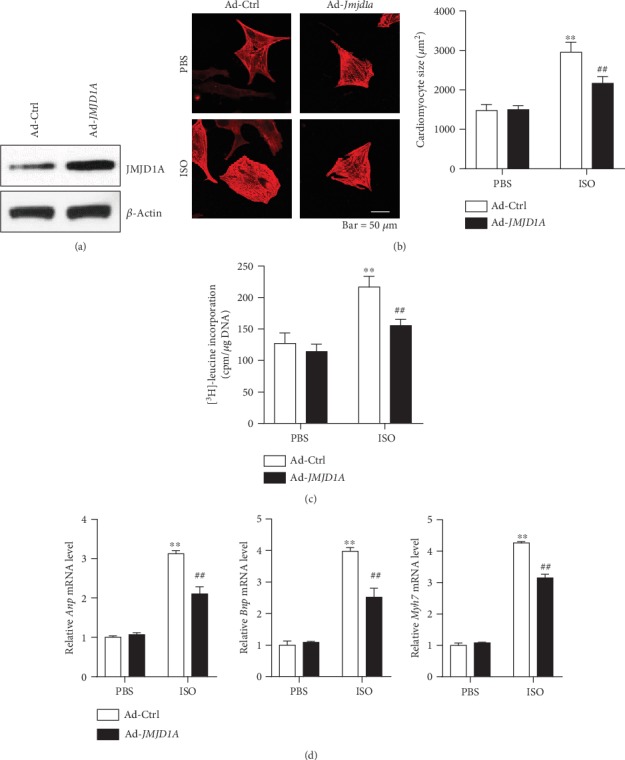
JMJD1A overexpression represses cardiomyocyte hypertrophy. (a) Representative western blot showing the expression of JMJD1A proteins in NRCMs infected with adenovirus carrying *Jmjd1a* or control adenovirus for 36 hours. (b) JMJD1A overexpression represses the ISO-induced increase in cardiomyocyte size. Representative images and quantitative results are shown. NRCMs were infected with indicated adenovirus for 24 hours and then treated with ISO (1 *μ*M) for 48 hours. ^∗∗^*p* < 0.01*vs.* PBS+Ad-Ctrl, ^##^*p* < 0.01*vs.* ISO+Ad-Ctrl. (c) JMJD1A overexpression represses ISO-induced protein synthesis in cardiomyocytes. NRCMs were infected with indicated adenovirus for 24 hours and then treated with ISO (1 *μ*M) for 48 hours. ^∗∗^*p* < 0.01*vs.* PBS+Ad-Ctrl, ^##^*p* < 0.01*vs.* ISO+Ad-Ctrl. (d) JMJD1A overexpression represses ISO-induced expression of hypertrophic fetal genes. NRCMs were infected with indicated adenovirus for 24 hours and then treated with ISO (1 *μ*M) for 48 hours. ^∗∗^*p* < 0.01*vs*. PBS+Ad-Ctrl, ^##^*p* < 0.01*vs.* ISO+Ad-Ctrl.

Next, we investigated whether JMJD1A overexpression could repress the development of cardiomyocyte hypertrophy. We overexpressed JMJD1A in NRCMs by infecting the cells with adenovirus carrying *Jmjd1a* (Ad-*Jmjd1a*, Figure 3(a)). We then treated the cells with ISO and analyzed the hypertrophic phenotype of cardiomyocytes. The results showed that JMJD1A overexpression repressed the effects of ISO-induced increase in cardiomyocyte size and protein synthesis (Figures 3(b) and 3(c)). Finally, we analyzed the effects of JMJD1A overexpression on ISO-induced expression of hypertrophic fetal genes and found that JMJD1A overexpression inhibited the expression of hypertrophic fetal genes (Figure 3(d)). Collectively, these findings demonstrated that JMJD1A inhibited cardiomyocyte hypertrophy.

### 3.3. ROS Is Involved in the Function of JMJD1A during Cardiomyocyte Hypertrophy

**Figure 4:**
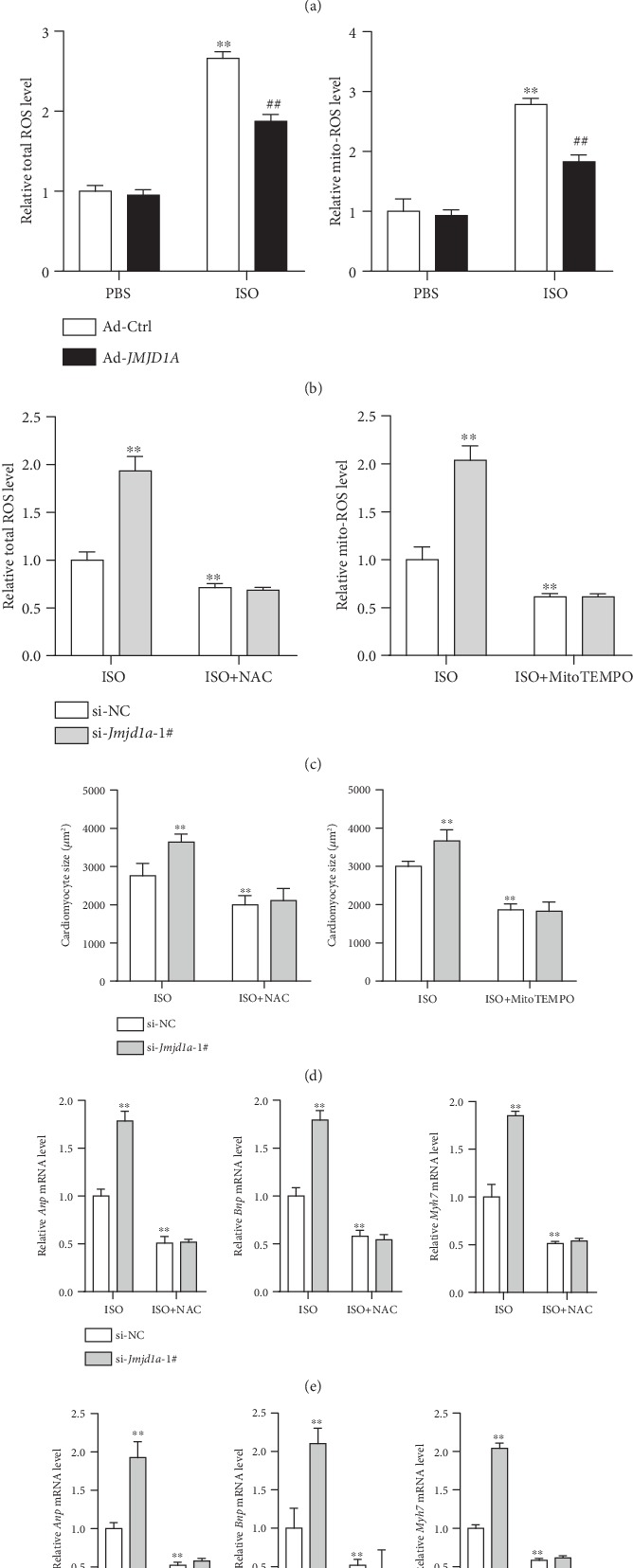
ROS contributes to the roles of JMJD1A in cardiomyocytes. (a) Jmjd1a knockdown promotes ISO-induced total cellular and mitochondrial ROS. NRCMs were infected with indicated adenovirus for 24 hours and then treated with ISO (1 *μ*M) for 12 hours. ^∗∗^*p* < 0.01*vs.* PBS+si-NC and ^##^*p* < 0.01*vs.* ISO+si-NC. (b) JMJD1A overexpression reduces ISO-induced total cellular and mitochondrial ROS. NRCMs were infected with indicated adenovirus for 24 hours and then treated with ISO (1 *μ*M) for 12 hours. ^∗∗^*p* < 0.01*vs.* PBS+si-NC and ^##^*p* < 0.01*vs.* ISO+si-NC. (c) NAC and MitoTEMPO repress *Jmjd1a* knockdown-mediated increase in total and mitochondrial ROS, respectively. NRCMs were transfected with indicated siRNA for 24 hours and then treated with ISO (1 *μ*M) in the presence of NAC (2 mM) or MitoTEMPO (50 nM) for 12 hours. ^∗∗^*p* < 0.01*vs.* PBS+si-NC and ^##^*p* < 0.01*vs.* ISO+si-NC. (d) NAC and MitoTEMPO repress *Jmjd1a* knockdown-mediated increase in cardiomyocyte size. NRCMs were transfected with indicated siRNA for 24 hours and then treated with ISO (1 *μ*M) in the presence of NAC (2 mM) or MitoTEMPO (50 nM) for 48 hours. ^∗∗^*p* < 0.01*vs.* PBS+si-NC. (e) NAC represses JMJD1A knockdown-mediated increase in expression of hypertrophic fetal genes. NRCMs were transfected with indicated siRNA for 24 hours and then treated with ISO (1 *μ*M) in the presence of NAC (2 mM) for 48 hours. ^∗∗^*p* < 0.01*vs.* PBS+si-NC. (f) MitoTEMPO represses the JMJD1A knockdown-mediated increase in the expression of hypertrophic fetal genes. NRCMs were transfected with indicated siRNA for 24 hours and then treated with ISO (1 *μ*M) in the presence of MitoTEMPO (50 nM) for 48 hours. ^∗∗^*p* < 0.01*vs.* PBS+si-NC.

Elevated total and mitochondrial ROS levels contribute to the development of cardiac hypertrophy [5]. To explore the potential mechanism underlying JMJD1A-mediated functions during cardiomyocyte hypertrophy, we tested the effects of JMJD1A on total cellular and mitochondrial ROS. Upon ISO treatment, the levels of total cellular and mitochondrial ROS were upregulated, which was facilitated by knocking down the expression of *Jmjd1a* with siRNA in NRCMs (Figure 4(a)). By contrast, adenovirus-mediated *Jmjd1a* overexpression repressed both total cellular and mitochondrial ROS levels (Figure 4(b)). Next, we examined whether the effects of JMJD1A on ROS contributed to its function during cardiomyocyte hypertrophy. Therefore, we used N-acetylcysteine (NAC) and MitoTEMPO to repress total cellular and mitochondrial ROS, respectively, which completely blocked the effects of *Jmjd1a* knockdown on ROS production (Figure 4(c)). We also observed that either NAC or MitoTEMPO treatment repressed cardiomyocyte hypertrophy and blocked the function of *Jmjd1a* knockdown as evidenced by cardiomyocyte size and the expression of hypertrophic fetal genes (Figures 4(d)–4(f)). Therefore, JMJD1A repressed the production of ROS, which was critically involved in the function of JMJD1A in cardiomyocyte hypertrophy.

### 3.4. JMJD1A Promotes the Expression of *Catalase* to Regulate ROS and Cardiomyocyte Hypertrophy

**Figure 5:**
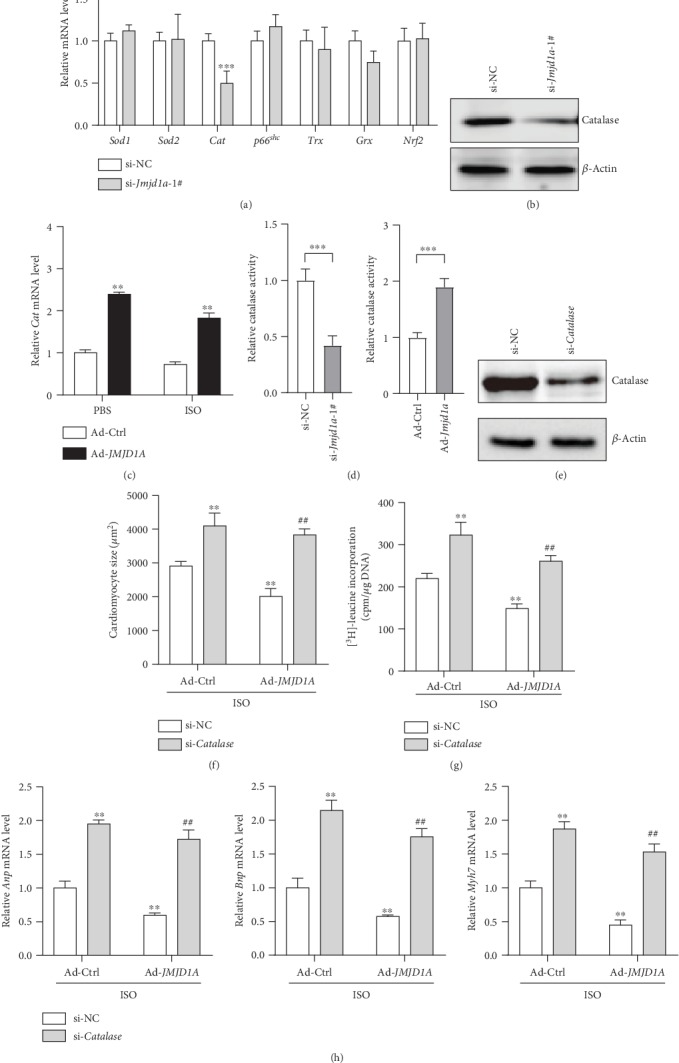
JMJD1A regulates catalase to modulate ROS and cardiomyocyte hypertrophy. (a) mRNA levels of oxidative genes and antioxidants in cardiomyocytes transfected with/without si-*Jmjd1a*. NRCMs were transfected with indicated siRNA for 36 hours. ^∗∗∗^*p* < 0.001*vs.* si-NC. (b) Protein levels of *Catalase* in cardiomyocytes transfected with/without si-*Jmjd1a*. NRCMs were transfected with indicated siRNA for 36 hours. (c) JMJD1A overexpression promotes the expression of *Catalase* under basal and ISO-induced stress. NRCMs were infected with indicated adenovirus for 24 hours and then treated with ISO (1 *μ*M) for an additional 24 hours. ^∗∗^*p* < 0.01*vs.* Ad-Ctrl. (d) JMJD1A controls Catalase activity. Cardiomyocytes were treated as in (a) or (c), then, catalase activity in cardiomyocytes was analyzed with a kit. (e) Representative western blot results showing *Catalase* knockdown in cardiomyocytes. NRCMs were transfected with indicated siRNA for 36 hours. (f) *Catalase* knockdown blocked JMJD1A-mediated repression in cardiomyocyte hypertrophy. NRCMs were infected with indicated adenovirus for 24 hours and then transfected with si-NC or si-*Catalase* for 24 hours, followed by ISO (1 *μ*M) treatment for an additional 48 hours. ^∗∗^*p* < 0.01*vs.* ISO+si-NC+Ad-Ctrl and ^##^*p* < 0.01*vs.* ISO+si-NC+Ad-*Jmjd1a*. (g) *Catalase* knockdown blocked JMJD1A-mediated repression of protein synthesis. The cells were treated as in (e). _∗∗_*p* < 0.01*vs.* ISO+si-NC+Ad-Ctrl and ^##^*p* < 0.01*vs.* ISO+si-NC+Ad-*Jmjd1a.* (h) *Catalase* knockdown blocks JMJD1A-mediated repression of the expression of hypertrophic fetal genes. The cells were treated as in ^∗∗^*p* < 0.01*vs.* ISO+si-NC+Ad-Ctrl and ^##^*p* < 0.01*vs.* ISO+si-NC+Ad-*Jmjd1a.*

We next investigated the mechanism underlying JMJD1A function in the regulation of ROS. We knocked down the expression of *Jmjd1a* in NRCMs and tested the enzymes in regulating cellular and mitochondrial ROS levels, including *Sod1*, *Sod2*, *Catalase*, *p66^shc^*, *Trx*, *Grx*, and *Nrf2.* The results showed that only the expression of *Catalase* was repressed by *Jmjd1a* knockdown, whereas the expression of the other factors was not affected by *Jmjd1a* (Figures 5(a) and 5(b)). To confirm the effect of *Jmjd1a* on *Catalase* expression, we overexpressed *Jmjd1a* in NRCMs and observed that *Jmjd1a* overexpression promoted the expression of *Catalase* under basal condition or under the stress induced by ISO (Figure 5(c)). In addition, we observed that *Jmjd1a* knockdown reduced catalase activity, whereas *Jmjd1a* overexpression led to oppose effects (Figure 5(d)), indicating that JMJD1A controlled Catalase expression and activity. Next, we investigated whether *Catalase* was critically involved in the development of cardiomyocyte hypertrophy. We knocked down the expression of *Catalase* with siRNA in NRCMs (Figure 5(e)). *Catalase* knockdown promoted the ISO-induced increase in cardiomyocyte size, protein synthesis, and expression of hypertrophic fetal genes. Interestingly, we observed that *Jmjd1a* overexpression was unable to regulate cardiomyocyte hypertrophy in NRCMs with *Catalase* deficiency (Figures 5(f) and 5(h)). These findings revealed that Catalase contributed to the function of JMJD1A in cardiomyocyte hypertrophy.

## 4. Discussion

Histone H3K9 demethylase JMJD1A is a signal-sensing scaffold that regulates acute chromatin dynamics via SWI/SNF association for thermogenesis [20]. JMJD1A demethylates H3K9me2 at the promoter regions of *Tcl1*, *Tcfcp2l1*, and *Zfp57* and positively regulates the expression of these pluripotency-associated genes [26]. The hypoxia-inducible epigenetic regulators JMJD1A and G9a provide a mechanistic link between angiogenesis and tumor growth [27]. In addition, JMJD1A modulates hepatic stellate cells activation and liver fibrosis by epigenetically regulating peroxisome proliferator-activated receptor *γ* [28]. JMJD1A interacts with the Myocardin factors to regulate smooth muscle cell differentiation marker gene expression [29]. Although a previous study has revealed the potential participation of JMJD1A in cardiac hypertrophy [22], the roles and underlying mechanisms of JMJD1A in cardiac hypertrophy across species are not fully understood. Here, we showed that JMJ1A participated in cardiac hypertrophy *via* targeting catalase to regulate oxidative stress. Our qRT-PCR and western blot evidence demonstrated that JMJD1A expression was downregulated in hypertrophic hearts. To further investigate the roles of JMJD1A, we knockdown the expression or overexpressed it in neonatal rat cardiomyocytes and demonstrated JMJD1A repressed cardiomyocyte hypertrophy induced by ISO. The function of JMJD1A was different from that of JMJD2A because the previous work of Zhang et al. demonstrated that JMJD2A promotes cardiac hypertrophy in response to hypertrophic stimuli in mice [15]. This finding implicates that different members of the JMJD family may have distinctive roles in cardiac hypertrophy.

Elevated total and mitochondrial ROS levels are the fundamental mechanisms underlying the development of cardiomyocyte hypertrophy *in vivo* and *in vitro* [5]. Indeed, we observed that JMJD1A was involved in the regulation of total and mitochondrial ROS levels under ISO-induced stress. Importantly, we used the antioxidant regent for total ROS (NAC) or mitochondrial ROS (MitoTEMPO) and observed that either NAC or MitoTEMPO treatment could repress the development of cardiomyocyte hypertrophy. These findings implicate that ROS could be a potential target for the treatment of cardiac hypertrophy. Importantly, we observed that the antioxidants NAC and MitoTEMPO blocked the effects of JMJD1A deficiency on ISO-induced increase in cardiomyocyte size, protein synthesis, and expression of hypertrophic fetal genes.

To further investigate the mechanism underlying the function of JMJD1A in the regulation of total and mitochondrial ROS, we explored the expression of enzymes in ROS production and elimination. We observed that Catalase was most important downstream of JMJD1A. Catalase is a pivotal antioxidant in cytoplasm and mitochondria [30]. Overexpression of *Catalase* led to the repression of total ROS and mice aging [31]. JMJD1A can promote the expression and enzymatic activity of Catalase in cardiomyocytes. We observed that *Catalase* was critically involved in the function of JMJD1A because *Catalase* knockdown blocked the protective function of JMJD1A during cardiac hypertrophy. However, the direct mechanism by which JMJD1A regulates Catalase expression is needed to explore in further study. In addition, other mechanisms may also participate in the function of JMJD1A. For instance, JMJD1A, under hypoxia, promotes the expression of the growth and differentiation factor 15 [32], which functions as a protective and antihypertrophic factor released from the myocardium in association with SMAD protein activation [33].

## 5. Conclusion

In conclusion, we demonstrate that the histone demethylase JMJD1A represses the development of cardiomyocyte hypertrophy by targeting catalase and oxidative stress.

## Data Availability

The data used to support the findings of this study are available from the corresponding author upon request.

## Supplementary Materials

Supplementary Materials "Primers used for quantitative PCR".
