# Supplementary material for: JMJD1A Represses the Development of Cardiomyocyte Hypertrophy by Regulating the Expression of Catalase
Source: Biomed Res Int. 2020 May 12;2020:5081323. doi: 10.1155/2020/5081323 (PMC7243027; doi:10.1155/2020/5081323)
Supplement: Supplementary Materials — "Primers used for quantitative PCR". [file 5081323.f1.docx]

**Supplementary data**

**Primers used for quantitative PCR.**

Human GAPDH forward: 5’-GGAGCGAGATCCCTCCAAAAT-3’

Human GAPDH reverse: 5’-GGCTGTTGTCATACTTCTCATGG-3’

Human ANP forward: 5’-TGCAGCTTCCTGTCAACACT-3’

Human ANP reverse: 5’-AGAGGCGAGGAAGTCACCAT-3’

Human BNP forward: 5’-TGGAAACGTCCGGGTTACAG-3’

Human BNP reverse: 5’-CTGATCCGGTCCATCTTCCT-3’

Human Myh7 forward: 5’-ACTGCCGAGACCGAGTATG-3’

Human Myh7 reverse: 5’-GCGATCCTTGAGGTTGTAGAGC-3’

Human JMJD1A forward: 5’-GTGCTCACGCTCGGAGAAA-3’

Human JMJD1A reverse: 5’-GTGGGAAACAGCTCGAATGGT-3’

Mouse Gapdh forward: 5’-AATGGATTTGGACGCATTGGT-3’

Mouse Gapdh reverse: 5’-TTTGCACTGGTACGTGTTGAT-3’

Mouse Anp forward: 5’-GTGCGGTGTCCAACACAGAT-3’

Mouse Anp reverse: 5’-TCCAATCCTGTCAATCCTACCC-3’

Mouse Bnp forward: 5’-GAGGTCACTCCTATCCTCTGG-3’

Mouse Bnp reverse: 5’-GCCATTTCCTCCGACTTTTCTC-3’

Mouse Myh7 forward: 5’-TTTCTGGCGACAAAGACAGGG-3’

Mouse Myh7 reverse: 5’-CTTGCCAAAACGGGAGGAGTT-3’

Mouse Jmjd1a forward: 5’-GTGGGGAAGCGATTCCTCAG-3’

Mouse Jmjd1a reverse: 5’-GTCTTTCTTAGTAACGTCGGTGT-3’

Rat Gapdh forward: 5’-ATGATTCTACCCACGGCAAG-3’

Rat Gapdh reverse: 5’-CTGGAAGATGGTGATGGGTT-3’

Rat Anp forward: 5’-ATCACCAAGGGCTTCTTCCT-3’

Rat Anp reverse: 5’-TGTTGGACACCGCACTGTAT-3’

Rat Bnp forward: 5’-GGACCAAGGCCCTACAAAAGAACT-3’

Rat Bnp reverse: 5’-CTAAAACAACCTCAGCCCGTCACA-3’

Rat Myh7 forward: 5’-CCCAACCCTAAGGATGCCTG-3’

Rat Myh7 reverse: 5’-TGTGTTTCTGCCTAAGGTGCT-3’

Rat Jmjd1a forward: 5’-ACTGTGAGGAGATTCCAGCG-3’

Rat Jmjd1a reverse: 5’-TGCTGTCTGTTGCTAGATGGG-3’

Rat Sod1 forward: 5’-AGGGCGTCATTCACTTCGAG-3’

Rat Sod1 reverse: 5’-CCTCTCTTCATCCGCTGGAC-3’

Rat Sod2 forward: 5’-CGGGGGCCATATCAATCACA-3’

Rat Sod2 reverse: 5’-GCCTCCAGCAACTCTCCTTT-3’

Rat Catalase forward: 5’-TTTTCACCGACGAGATGGCA-3’

Rat Catalase reverse: 5’-CCCACAAGGTCCCAGTTACC-3’

Rat p66shc forward: 5’-TAAGCAGACAGTTGCGTGGT-3’

Rat p66shc reverse: 5’-TGAAGTCAAGGGCTCGCATT-3’

Rat Trx forward: 5’-GTGGTGTGGACCTTGCAAAA-3’

Rat Trx reverse: 5’-GGAAGGTCGGCATGCATTTG-3’

Rat Grx forward: 5’-CAAACCCACCTGCCCCTATT-3’

Rat Grx reverse: 5’-GGAACTGTTCTTGCTCCGGT-3’

Rat Nrf2 forward: 5’-TTTGTAGATGACCATGAGTCGC-3’

Rat Nrf2 reverse: 5’-GCCAAACTTGCTCCATGTCC-3’
